# Exploring Geographic Variability in Cancer Prevalence in Eastern Morocco: A Retrospective Study over Eight Years

**DOI:** 10.1371/journal.pone.0151987

**Published:** 2016-03-21

**Authors:** Manal Elidrissi Errahhali, Mounia Elidrissi Errahhali, Naima Abda, Mohammed Bellaoui

**Affiliations:** Medical Biology Unit, Faculty of Medicine and Pharmacy of Oujda, University Mohammed the First, Oujda, Morocco; Rudjer Boskovic Institute, CROATIA

## Abstract

**Background:**

Malignant diseases have been believed to be more common in some areas of Eastern Morocco, but until now, cancer patterns have not been reported for this region. In this paper we present for the first time the cancer prevalence analysis in Eastern Morocco.

**Methods:**

Cross-sectional study carried out among all patients diagnosed and/or treated with cancer at the Hassan II Regional Oncology Center (ROC) since it was established in October 2005 until December 2012. The ROC is the only hospital specialized in cancer care in Eastern Morocco.

**Results:**

A total of 8,508 cases of cancer were registered among residents in Eastern Morocco, with a female to male ratio of 2.1. The mean age at diagnosis was 53.9 ± 15.2 years (median age = 53 years). Thus, unlike in Western countries, cancer in Eastern Morocco afflicts younger population. The areas of Eastern Morocco did not differ significantly by mean age at diagnosis (*p* = 0.061). However, these regions differed significantly by sex ratio (*p* < 0.001). The highest sex ratio was observed in Figuig, with a female to male ratio of 3.1 (75.4% of the registered case were females), followed respectively by Taourirt, Oujda-Angad, Berkane, Nador-Driouch and Jerada. Clear variation in the distribution of cancer types between areas of Eastern Morocco was observed, both in males and females (p < 0.001). Furthermore, the areas of Eastern Morocco differed significantly by cancer prevalence (p < 0.001). The highest age-standardized five-year prevalence proportion was observed in Oujda-Angad with 420.2 per 100,000, followed respectively by Berkane (311.4), Jerada (287.8), Taourirt (269.3), Nador-Driouch (213.6) and Figuig (194.4). Trends in the five-year prevalence proportions decreased in Oujda-Angad, Berkane and Jerada throughout the study period, while an increasing trend was observed in Nador-Driouch, Taourirt and Figuig.

**Conclusions:**

For the first time, our study presents the pattern and distribution of diagnosed cancers in Eastern Morocco. Our study illustrates substantial differences in cancer patterns between areas of Eastern Morocco. These findings are important for cancer control and highlight the need to develop program aiming at controlling and preventing the spread of major cancer sites in Eastern Morocco, particularly in areas with increased cancer prevalence rates.

## Introduction

According to WHO, global cancer prevalence is growing at “alarming pace” [1]. In Morocco, cancer is one of the major health problems and it is the second leading cause of mortality after cardiovascular diseases with 10.7% of all deaths [2]. However, there is no national cancer reporting system in Morocco. There are only two population-based cancer registries at the present time, the Rabat and Casablanca registries [3–5]. However, in order to develop an effective national program for cancer control and prevention, it is essential to study the burden of cancer in each area of Morocco.

**Fig 1:**
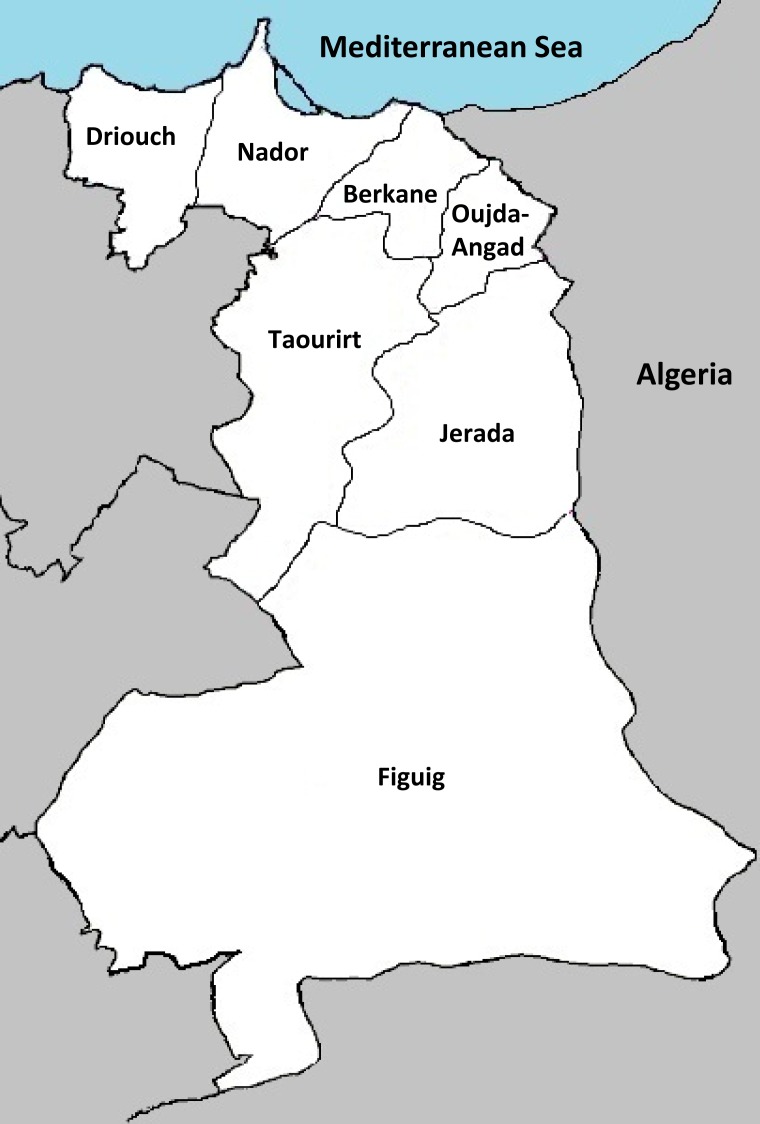
Schematic representation of Eastern Morocco region. Adapted from the High Commission for Planning 2012 document, see http://www.hcp.ma/region-oriental/docs/monographieFr2012/monographie%202012.pdf.

Eastern Morocco is the third largest region in Morocco, but there is still no population-based cancer registry until now. Located at the border between Morocco and Algeria and close to Europe, this region has been a hot spot throughout the history. Eastern Morocco is located in the north east of the kingdom of Morocco, and extends on an area of 82,820 km2, which is equivalent of 11.6% of the national territory. According to the administrative division of 2009, Eastern Morocco is divided into one prefecture (Oujda-Angad) and six provinces (Nador, Driouch, Berkane, Taourirt, Jerada and Figuig) (Fig 1). According to the High Commission for Planning (HCP), Eastern Morocco had a population of over 2 million in 2013, which is equivalent to 6.2% of the total population of the Kingdom. The population is mainly urban (67% vs. 33% rural) and young, nearly 6 out of 10 people are under 30 years [6].

Determination of cancer prevalence is an important measure to study the burden of cancer [7–13]. Therefore, in this retrospective study, we report the geographic variability in cancer prevalence and patterns in Eastern Moroco over a period of eight years between 2005 and 2012.

## Materials and Methods

### Design of the study

This is a cross-sectional study carried out among all hospitalized or monitored patients for cancer at the Hassan II Regional Oncology Center (ROC) since October 2005 until December 2012. The ROCwas establishedin October 2005 andisthe only hospital specialized in cancer care in Eastern Morocco and it includes several units, such as surgery, chemotherapy, and radiotherapy.

### Study population

The study population consists of all cancer cases that were registered at the ROCsince it was established in October 2005 until December 2012. The majority (98%) of the cases were microscopically verified. The registrations were considered microscopically verified when the diagnosis was based on malignant histological or cytological reports. We excluded from the study patients for whom the proof of cancer could not be made or the medical file is incomplete or unexploitable. The borderline tumors and cases of intraepithelial neoplasia were also excluded from the study. Patients who do not reside in Eastern Morocco or for whom the place of residence was not specified were also excluded from the analysis.

### Data collection and cancer classification

The data were collected from patient medical records, pathology records and admission records. We followed the registration rules, defined by the International Agency for Research on Cancer (IARC). The registred cases were coded according to the third edition of the International Classification of Diseases for Oncology (ICD-O-3) [14]. For tabulation of results, these were converted to the 10th revision of the ICD [15]. A form has been used for collecting information recorded on each case, such as the record number, name and surname of the patient, gender, age, place of residence,years of diagnosis, cancer site, and histological type.

### Analysis

Data collection was performed on Excel.Statistical analysis was performed using SPSS software version 21.0. Initially, descriptive statistics were used to describe the distribution of cancer by age, sex and area. To evaluate differences between each area we used Chi2 to evaluate categorical variables (sex) and one-way analysis of variance (ANOVA) for continuous variables (age). The results were considered significant when *p* (degree of significance) is less than 0.05, very significant when *p*< 0.01 and highly significant when *p*< 0.001.

Total prevalence proportions (per 100,000) were calculated for each area of Eastern Morocco by dividing prevalence counts by the corresponding population size and multiplying by 100,000 [16]. Five-year prevalence was estimated by counting the number of cases diagnosed in the previous five years and still alive at a given index date [11]. For example, five-year prevalence for 2012 was estimated by counting the number of cases diagnosed between 2008 and 2012 who were still alive at the end of 2012 [11]. We calculated five-year prevalence at three time points for which data were available (2010, 2011, and 2012).The proportions were expressed per 100,000 person [17]. Age-standardized five-year prevalence proportions (per 100,000) were calculated by the direct method, using the world standard population as previously described [17–19]. The age-standardized rate is a summary of the individual age-specific rates using an external population called a standard population. This is the prevalencethat would be observed if the population had the age structure of the standard population, and corresponds to the prevalence rate in the standard population [17–19]. National population censuses are conducted in Morocco every 10 years, and the High Commission for Planning provides estimates of the growth rate of the Moroccan population for each year. In this paper, the census conducted in 2004 was used to elaborate the estimates of the population of Eastern Morocco during the period from 2006 to 2012.The annual percent change (APC) was calculated as previously described [17, 20] using the formula: APC = [exp (B) - 1] × 100, where B is the parameter estimate obtained on fitting period of eventas a continuous variable to the logarithm of the rate.

### Ethical approval and authorization for personal data processing

In this study, obtaining informed consent was not possible. So, we were granted a waiver of consent by the Ethical Review Committee, and patient records/information was anonymized and de-identified prior to analysis. Our study protocol was ethically approved by the Ethical Review Committee for Biomedical Research of the Faculty of Medicine and Pharmacy of Casablanca under the number 41/14. The authorization for personal data processing was obtained from the National Commission of control of Personal Data Protection under the number A-RS-280/2014).

## Results

9,299 prevalent cases of cancer were registered in the Regional Oncology Centersince it was established in October 2005 until December 2012. Of these, 8,508 cases were residents of Eastern Morocco and were considered in the analysis. 5,779 cases were women and 2,729 men, with a female to male ratio of 2.1. The mean age at diagnosis was 53.9 ± 15.2 years (median age = 53 years) (Table 1).

**Table 1:** Mean age at diagnosis and sex ratio for all cancers combined by area of Eastern Morocco, October 2005–December 2012.

Regions	All areas	Oujda-Angad	Berkane	Nador-Driouch	Jerada	Taourirt	Figuig	*P-value*
**(No. of cases)**	(8508)	(3434)	(1418)	(2086)	(464)	(789)	(317)	
**Mean age (years) ±SD**	53.9 ± 15.2	54.4 ±15.5	54.1 ± 14.7	53 ± 14.4	54.2 ± 15.9	53.8 ± 16.1	53.9 ±16.2	0.061
**% F (F/M)**	67.9 (2.1)	69.2 (2.3)	66.8 (2.0)	65.3 (1.9)	64.4 (1.9)	70.3 (2.4)	75.4 (3.1)	< 0.001

% F: percentage of female cases among all registered cases; F/M: female to male ratio

The sex ratio and the mean age at diagnosis were calculated for each area of Eastern Morocco and the results are shown in Table 1.The areas of Easten Morocco did not differ significantly by mean age at diagnosis (*p* = 0.061). However, these regions differed significantly by sex ratio (*p*< 0.001). The highest sex ratio was observed in Figuig, with a female to male ratio (F/M) of 3.1 (75.4% of the registred case were females), followed respectively by Taourirt with a F/M of 2.4, Oujda-Angadwith a F/M of 2.3, Berkane with a F/M of 2.0, Nador-Driouch and Jeradawith a F/M of 1.9 each (Table 1).

**Fig 2:**
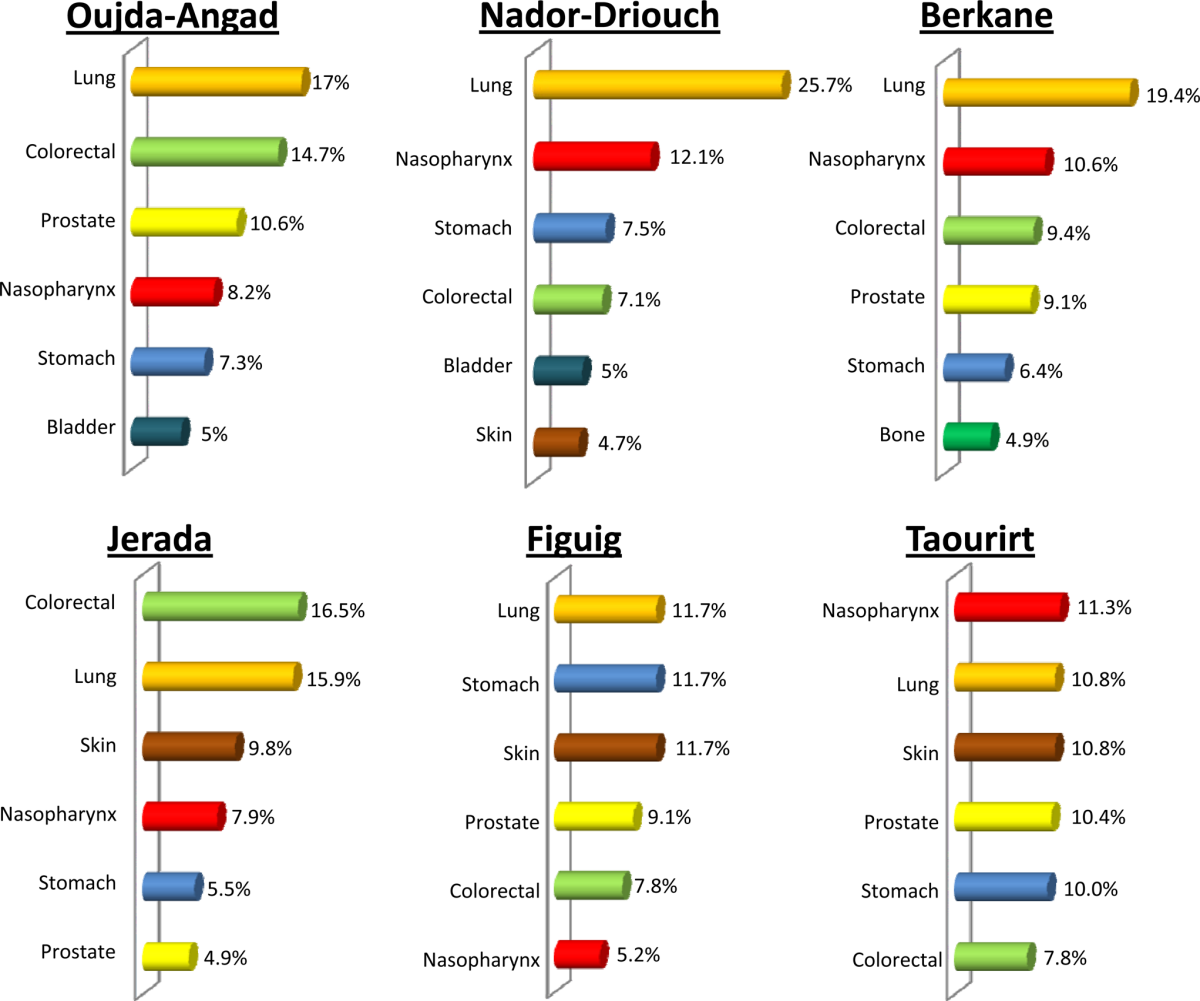
Distribution pattern of major cancer sites in males by area of Eastern Morocco, October 2005–December 2012.

**Fig 3:**
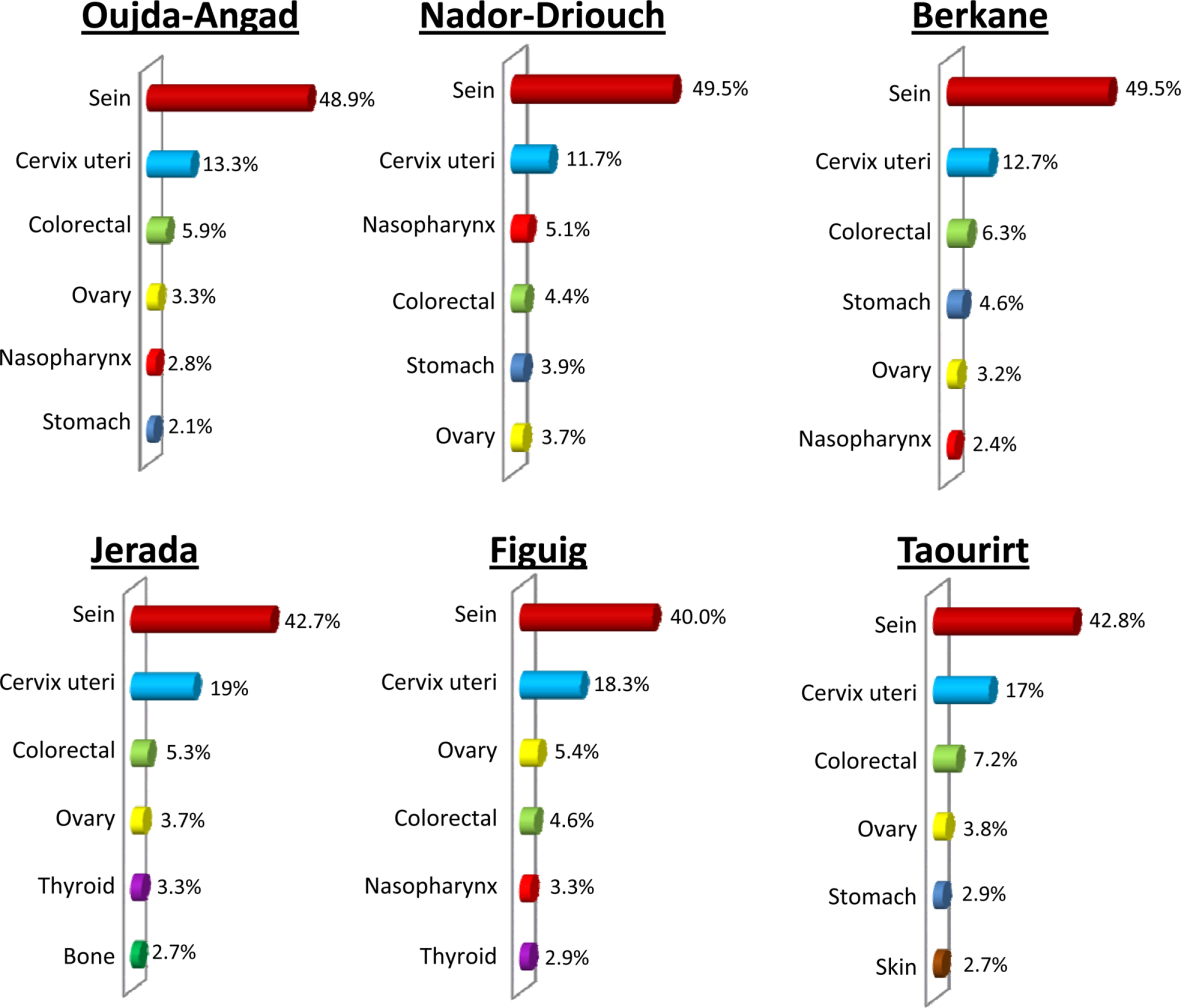
Distribution pattern of major cancer sites in females by area of Eastern Morocco, October 2005–December 2012.

Clear variation in the distribution of cancer types between areas of Eastern Morocco was observed, both in males and females (p< 0.001). For example, in males, lung cancer is the most frequent cancer type in Oujda-Angad, Nador-Driouch, Berkane and Figuig (Fig 2; S1 Table). However, this cancer ranked second in Jerada and Taourirt (Fig 2; S1 Table). Similarly, colon-rectum cancer ranked first in Jerada, second in Oujda-Angad, third in Berkane, fourth in Nador-Driouch, fifth in Figuig and sixth in Taourirt (Fig 2; S1 Table). In the same way, nasopharynx cancer ranked first in Taourirt, second in Nador-Driouch and Berkane, fourth in Oujda-Angad and Jerada, and sixth in Figuig (Fig 2; S1 Table). In women, breast cancer was the most frequent malignancy, followed by cervix uteri cancer in all areas of Eastern Morocco (Fig 3; S2 Table). However, the frequencies of these cancers varied between areas. Indeed, the highest frequency of breast cancer was observed in Nador-Driouch and Berkane (49.5%), followed respectively by Oujda-Angad (48.9%), Taourirt (42.8%), Jerada (42.7%), and Figuig (40.0%) (Fig 3; S2 Table).

Geographic variation in cancer prevalence during the period 2006–2012 was also carried out and the result is shown in Table 2.The areas of Eastern Morocco differed significantly by cancer prevalence proportions (p < 0.001). The highest totalprevalence proportion from 2006 to 2012 was observed in Oujda-Angad (660.8 per 100,000), followed respectively by Berkane (497 per 100,000), Jerada (402.7 per 100,000), Taourirt (355.6 per 100,000), Nador-Driouch (275 per 100,000) and Figuig (236.4 per 100,000) (Table 2). Similarly, the highest five-year prevalence proportions during the study period were observed in Oujda-Angad followed respectively by Berkane, Jerada, Taourirt, Nador-Driouch and finally Figuig (Table 2).

**Table 2:** Total and five-year prevalence proportions by area of Eastern Morocco.

Period		All areas	Oujda-Angad	Berkane	Nador-Driouch	Jerada	Taourirt	Figuig
**Total prevalence 2006–2012**	No. of Cases	8248	3284	1391	2053	447	768	305
	Proportion (100,000)	416,7	660.8	497	275	402.7	355.6	236.4
**Five year prevalence 2006–2010**	No. of Cases	5693	2305	994	1344	328	512	210
	Proportion (100,000)	289.4	467.5	357.6	180.8	298.2	239.3	162.8
**Five year prevalence 2007–2011**	No. of Cases	5607	2279	947	1395	294	477	215
	Proportion (100,000)	283.2	458.6	338.2	186.8	264.9	220.8	166.7
**Five year prevalence 2008–2012**	No. of Cases	5833	2315	947	1510	296	533	232
	Proportion (100,000)	292.9	462.1	336.1	201.4	264.3	244.5	179.8

**Fig 4:**
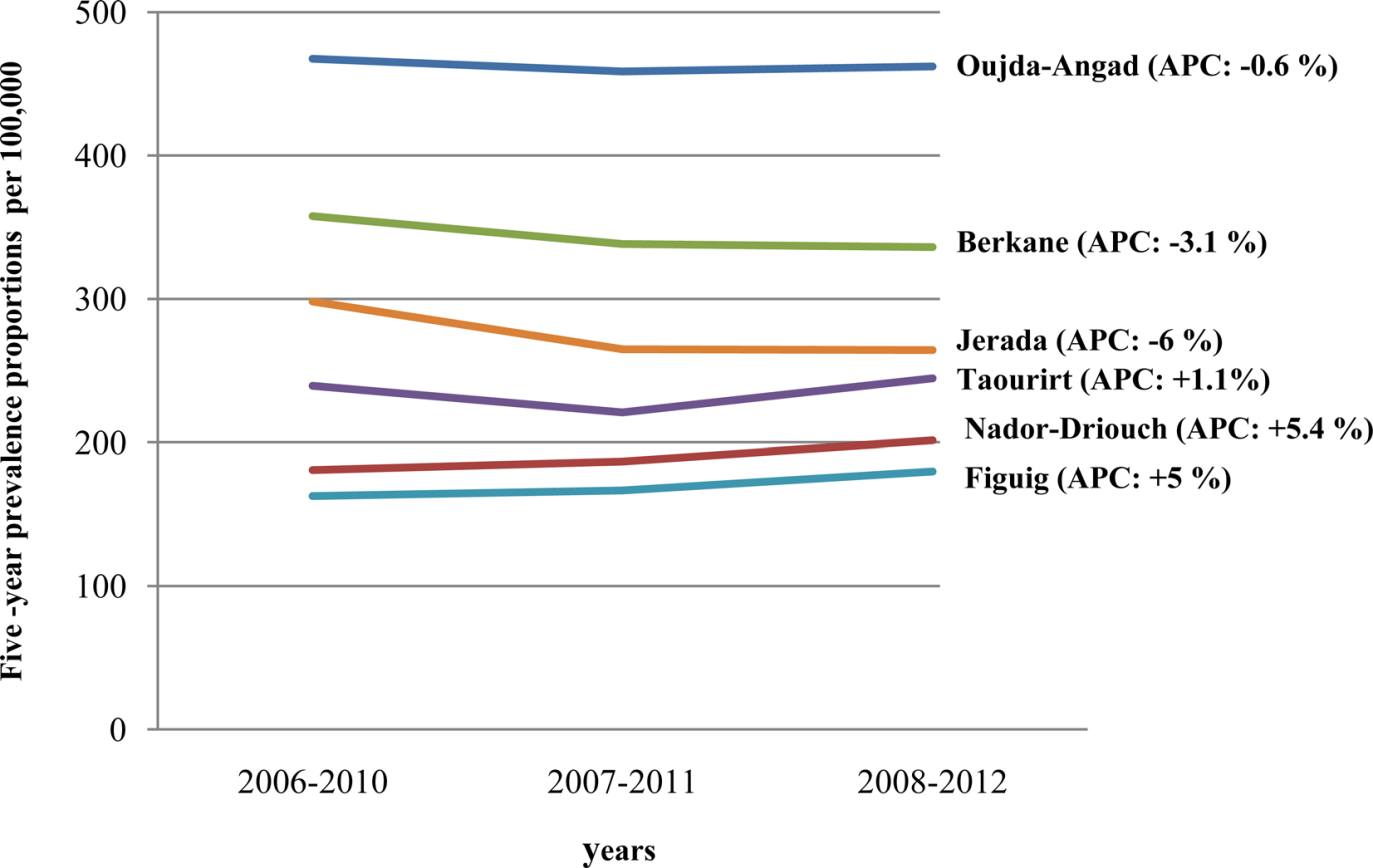
Trends in the five-year prevalence proportions by area of Eastern Morocco.

Trends in the five-year prevalence proportions in the study period were noticeably different between areas of Eastern Morocco (Fig 4). The five-year cancer prevalence proportionin Oujda-Angad slightly decreased,at an APC of 0.6% (Fig 4). In Berkane and Jerada, the five-year prevalence proportion significantly decreased,at an APC of 3.1% and 6%, respectively (Fig 4). In contrast, there is a marked increase in the five-year prevalence proportion in Nador-Driouch and Figuig by 5.4% and5%, respectively (Fig 4). Similarly, the five-year cancer prevalence proportion increased in Taourirt by 1.1% (Fig 4).

## Discussion

Cancer is one of the major health problems worldwide [21–23]. Cancer patterns can display great variation between different ethnic populations and areas in the same country[7–13, 24, 25].

Our study showed that there is no significant difference in the mean age at diagnosis between areas of Eastern Morocco. We found that cancer afflict adult population in all areas of Eastern Morocco, with an overall mean age at diagnosis of 53.9 ± 15.2 years (median age = 53 years).In European countries, the largest proportion of all prevalent cases were aged 65 years of age or over [10]. Thus, unlike Western countries, cancer in Eastern Morocco afflicts younger population. This young age phenomenon observed is probably due to the younger population [6]. However, multiple factors may be involved, including genetic and environmental factors. Further studies are needed to confirm the involvement of these factors in the occurrence of cancer at a younger age in Eastern Morocco.

We found that in Eastern Morocco 67.9% of all prevalent cancer cases were females(female to male ratio of 2.1). This high female to male ratio cannot be explained by the structure of the population of Eastern Morocco which showed a sex ratio of female to male of 1.06 during the study period 2006–2012 [6]. This suggests that in Eastern Morocco, women are more afflicted by cancer than men. This female predominance is smilar to that reported in European countries where 61% of all prevalent cancer cases were females[10]. However, in China cancer prevalence estimates were only slithly higher for females compared to males, with a female to male ratio of 1.04[13]. The higher prevalence of cancer in females is most likely attributed to the high prevalence of gynecological and breast cancers. Typically female cancers are usually more common in developing countries as opposed to developed countries [26]. More importantly, the areas of Eastern Moroco differed significantly by sex ratio (*p*< 0.001). The proportion of prevalent cancer cases that were women ranged from 64.4% to 75.4%.The highest sex ratio was observed in Figuig, followed respectively by Taourirt, Oujda-Angad, Berkane, Nador-Driouch and Jerada.A similar geographic variability in sex ratio was observed in Europe where the proportion of prevalent cancer cases that were women ranged from 53% in Spain to 71% in Poland [10]. Further research is needed to understand the sex ratio difference observed betweenareas of Eastern Morocco.

Remarkable geographic variation in the distribution of cancer types was observed in areas of Eastern Morocco. This variation could be due to differences in the prevalence of risk factors. For example, variation in lung cancer prevalence could be attribuated to differences in smoking prevalence among areas, since the association between lung cancer and smoking is well established [27]. Several other risk factors could be involved in this geographic variation in cancer prevalence, like differences in diet, physical activity, lifestyle, genetic factors, and infections prevalence with virus such as human papillomavirus (HPV) or bacteria such as Helicobacter pylori[28–31].

The geographic variation in cancer prevalence could be also due to differencesin the use of screening tests. For exmaple, variation in prostate cancer prevalence could be attribuated to differences in prostate-specific antigen (PSA) testing prevalence among areas[32, 33]. Likewise, variation in breast cancer prevalence could be partly due to differences in mammography prevalence between areas[34–36]. Therefore, the involvement of the differences inthe prevalence of risk factors, use of screening tests, and diagnostic practices must be studied to understand the geographic variation in cancer prevalence observed in Esatern Morocco.

Malignant diseases have been believed to be more common in some areas of Eastern Morocco, but until now, cancer prevalence and incidence have not been reported for this region. Thus, our study compared the prevalence of cancer between the areas of Eastern Morocco during the period 2006–2012. The highest total and five-year prevalence proportions were observed in Oujda-Angad followed respectively by Berkane, Jerada, Taourirt, Nador-Driouch and finally Figuig. Furtermore, the areas of Eastern Morocco differed significantly by cancer prevalence proportions. For example, the five-year prevalence proportions from 2008 to 2012 varied between 462.1 per 100,000 in Oujda-Angad and 179.8 per 100,000 in Figuig. Such finding is not very far from that reported in north African countries, where the estimated five-year prevalenceproportions from 2008 to 2012 varied between 372.2 per 100,000 in Egypt and 282.4 per 100,000 in Libya [7, 37, 38] (Table 3).

**Table 3:** Five-year prevalence proportions for all cancers in area of Eastern Morocco as compared to other North African countries.

		Five year prevalence 2008–2012	
Country		Numbers	Proportion (100,000)
Eastern Morocco^a^		5833	292.9
	Oujda-Angad	2315	462.1
	Berkane	947	336.1
	Nador-Driouch	1510	201.4
	Jerada	296	264.3
	Taourirt	533	244.5
	Figuig	232	179.8
North African countries^b^		463898	323.9
	Morocco	82503	336.1
	Algeria	86382	322.7
	Tunisia	25513	310.1
	Libya	12626	282.4
	Egypt	215486	372.2

^a^this study

^b^Estimated five-year prevalence from the GLOBOCAN project, see http://globocan.iarc.fr

Large differences in cancer prevalence were also observed between Nordic countries[39, 40]. Indeed, the age-standardized five-year prevalence proportions for all cancers varied between 1908.6 per 100,000 in Danmark and 1435.2 per 100,000 in Iceland (Table 4) [39, 40]. It is worth noting that the age-standardized five-year prevalence proportions observed in this study were much lower than those reported in Nordic countries (Table 4) [39, 40]. Large differences in cancer prevalence were also observed between European countries as well as between United States[8, 10, 25].

**Table 4:** Age-standardized five-year prevalence proportions for all cancers in area of Eastern Morocco as compared to other countries.

		Five year prevalence 2008–2012	
Country		Numbers	Proportion (100,000)
Eastern Morocco^a^		5651	292.9
	Oujda-Angad	2244	420.2
	Berkane	913	311.4
	Nador-Driouch	1463	213.6
	Jerada	289	287.8
	Taourirt	520	269.3
	Figuig	222	194.4
Nordic countries^b^		451163	1738.4
	Danmark	106841	1908.6
	Finland	87126	1605.5
	Iceland	4622	1435.2
	Norway	85854	1701.4
	Sweden	166720	1744.0

^a^this study

^b^Age-standardized five-year prevalence from the NORDCAN project, see http://www-dep.iarc.fr

Trends in the five-year prevalence proportions were also analyzed during the study period. We found a noticeable difference in cancer prevalence between areas of Eastern Morocco. Indeed, the five-year prevalence proportions decreased in Oujda-Angad, Berkane and Jerada. However, there was a moderate increase in the five-year prevalence proportions in Taourirt, and a marked increase in the regions of Nador-Driouch, and Figuig. It is worth noting that the geographic variation in cancer prevalence presented in this study may be influenced by differences in the accessibility to care at the Regional Oncology Center of Oujda. Moreover, cancer prevalence is a function of the incidence, survival and population demographics [9]. Therefore, our data is a very first step to understand cancer prevalence in Eastern Morocco and further extensive studies are necessary to better understand the reasons for the geographic variability in cancer prevalence among areas of Eastern Morocco.

## Conclusion

This study of a large number of patients diagnosed with cancer is a very first step to understand the geographic variability in cancer prevalence in Eastern Morocco. The areas of Eastern Morocco differed significantly by the distribution pattern of major cancer sites, sex ratio, and trends in cancer prevalence. These findings justify the need to establish a regional cancer registry as a first step to better understand this geographic variability in cancer prevalence in Eastern Morocco. The findings highlight also the need to develop program aiming at controlling and preventing the spread of major cancer sites in Eastern Morocco, particularly in areas with increased cancer prevalence rates.

## Supporting Information

S1 Table Distribution pattern of cancer in males by area of Eastern Morocco, October 2005-December 2012. (PDF)

S2 Table Distribution pattern of cancer in females by area of Eastern Morocco, October 2005-December 2012. (PDF)
