# Correction: Exploring Geographic Variability in Cancer Prevalence in Eastern Morocco: a Retrospective Study over Eight Years

**DOI:** 10.1371/journal.pone.0155200

**Published:** 2016-05-04

**Authors:** Manal Elidrissi Errahhali, Mounia Elidrissi Errahhali, Naima Abda, Mohammed Bellaoui

Fig 3 is incorrectly labelled in the published article. The word “Sein” should be replaced with “Breast” throughout the figure. The correct Fig 3 can be downloaded here.

**Fig 3:**
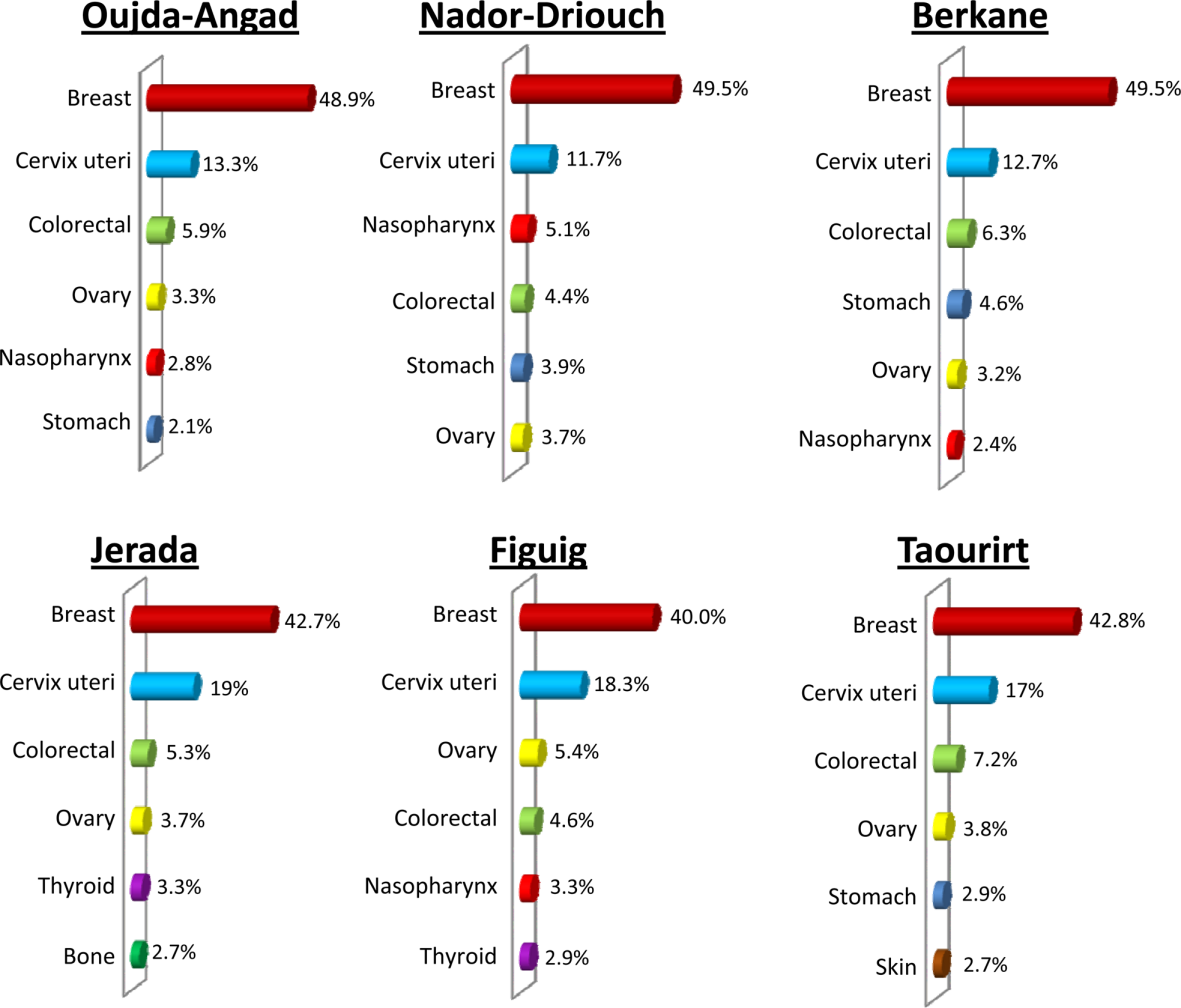
Distribution pattern of major cancer sites in females by area of Eastern Morocco, October 2005–December 2012.
