# Supplementary material for: Exploring Geographic Variability in Cancer Prevalence in Eastern Morocco: A Retrospective Study over Eight Years
Source: PLoS One. 2016 Mar 21;11(3):e0151987. doi: 10.1371/journal.pone.0151987 (PMC4801360; doi:10.1371/journal.pone.0151987)
Supplement: S1 Table — (PDF) [file pone.0151987.s001.pdf]

S1 Table. Distribution pattern of cancer in males by area of Eastern Morocco, October 2005-December 2012.

Site	ICD-10	Oujda-Angad %	Berkane %	Nador-Driouch %	Jerada %	Taourirt %	Figuig %
**Lung**	C34	17	19.4	25.7	15.9	10.8	11.7
**Nasopharynx**	C11	8.2	10.6	12.1	7.9	11.3	5.2
**Larynx**	C32	3.3	3.2	3.5	1.2	2.6	2.6
**Colorectal**	C18-C20	14.7	9.4	7.1	16.5	7.8	7.8
**Stomach**	C16	7.3	6.4	7.5	5.5	10	11.7
**Liver**	C22	1.7	1.5	1.8	1.8	2.2	5.2
**Prostate**	C61	10.6	9.1	4.2	4.9	10.4	9.1
**Bladder**	C67	5	4.7	5	3.7	4.8	0
**Skin**	C44	4	4	4.7	9.8	10.8	11.7
**Brain**	C70-72	2.7	3.8	4.6	4.9	4.3	3.9
**Bone**	C40-41	2.7	4.9	2.8	4.9	4.3	0
**Breast**	C50	2	2.6	1.4	3	2.6	3.9
**Thyroid**	C73	0.9	0.6	0.8	3	0.4	1.3
**Others**	-	20	19.8	19	17.1	17.7	26
**All sites**	**All**	**100**	**100**	**100**	**100**	**100**	**100**

Nom du document : S1_Table
Répertoire : C:\Documents and Settings\PC\Mes documents
Modèle : C:\Documents and Settings\PC\Application Data\Microsoft\Templates\Normal.dotm
Titre :
Sujet :
Auteur : User
Mots clés :
Commentaires :
Date de création : 09/03/2016 19:13:00
N° de révision : 3
Dernier enregistr. le : 09/03/2016 19:14:00
Dernier enregistrement par : fmpo
Temps total d'édition : 1 Minute
Dernière impression sur : 10/03/2016 08:21:00
Tel qu'à la dernière impression
Nombre de pages : 1
Nombre de mots : 114 (approx.)
Nombre de caractères : 629 (approx.)
