# Supplementary material for: Exploring Geographic Variability in Cancer Prevalence in Eastern Morocco: A Retrospective Study over Eight Years
Source: PLoS One. 2016 Mar 21;11(3):e0151987. doi: 10.1371/journal.pone.0151987 (PMC4801360; doi:10.1371/journal.pone.0151987)
Supplement: S2 Table — (PDF) [file pone.0151987.s002.pdf]

S2 Table. Distribution pattern of cancer in females by area of Eastern Morocco, October 2005-December 2012.

Site	ICD-10	Oujda-Angad %	Berkane %	Nador-Driouch %	Jerada %	Taourirt %	Figuig %
**Breast**	C50	48.9	49.5	49.5	42.7	42.8	40
**Cervix uteri**	C53	13.3	12.7	11.7	19	17	18.3
**Ovary**	C56	3.3	3.2	3.7	3.7	3.8	5.4
**Colorectal**	C18-C20	5.9	6.3	4.4	5.3	7.2	4.6
**Stomach**	C16	2.1	4.6	3.9	2.3	2.9	1.3
**Liver**	C22	0.9	0.8	1	1	1.8	1.7
**Lung**	C34	1.6	1.7	1.8	1.3	1.6	0.8
**Nasopharynx**	C11	2.8	2.4	5.1	2.3	1.6	3.3
**Larynx**	C32	0.1	0.3	0.3	0.3	0	0
**Skin**	C44	2.1	1.7	1.8	1	2.7	2.5
**Brain**	C70-72	1.3	1.8	1.9	1.3	0.4	0.8
**Bone**	C40-41	1.4	1.6	1.5	2.7	1.8	0.4
**Thyroid**	C73	1.9	1.7	1.2	3.3	2	2.9
**Bladder**	C67	0.7	0.6	0.1	0.7	0.2	2.1
**Others**	-	13.7	11.1	12.2	13	14.3	15.8
**All sites**	**All**	**100**	**100**	**100**	**100**	**100**	**100**

Nom du document : S2_Table
Répertoire : C:\Documents and Settings\PC\Mes documents
Modèle : C:\Documents and Settings\PC\Application Data\Microsoft\Templates\Normal.dotm
Titre :
Sujet :
Auteur : User
Mots clés :
Commentaires :
Date de création : 09/03/2016 19:13:00
N° de révision : 3
Dernier enregistr. le : 09/03/2016 19:14:00
Dernier enregistrement par : fmpo
Temps total d'édition : 1 Minute
Dernière impression sur : 10/03/2016 08:22:00
Tel qu'à la dernière impression
Nombre de pages : 1
Nombre de mots : 121 (approx.)
Nombre de caractères : 666 (approx.)
